# Challenges in and lessons learned during the implementation of the 1-3-7 malaria surveillance and response strategy in China: a qualitative study

**DOI:** 10.1186/s40249-016-0188-8

**Published:** 2016-10-05

**Authors:** Guangyu Lu, Yaobao Liu, Claudia Beiersmann, Yu Feng, Jun Cao, Olaf Müller

**Affiliations:** 1 Department of Preventive Medicine, Medical College of Yangzhou University, Yangzhou University, 225001 Yangzhou, China; 2 Institute of Public Health, Medical School, The Ruprecht-Karls-Universität Heidelberg, INF 324, 69120 Heidelberg, Germany; 3 Key Laboratory of National Health and Family Planning Commission on Parasitic Disease Control and Prevention, Jiangsu Provincial Key Laboratory on Parasite and Vector Control Technology, Jiangsu Institute of Parasitic Diseases, Wuxi, China; 4 Gansu Provincial Center for Disease Control and Prevention, Lanzhou, China; 5 Public Health Research Center, Jiangnan University, Wuxi, China

**Keywords:** Malaria, 1-3-7 surveillance and response strategy, China, Qualitative research

## Abstract

**Background:**

China has made great progress in malaria control over the last century and now aims to eliminate malaria by 2020. In 2012, the country launched its 1-3-7 surveillance and response strategy for malaria elimination. The strategy involves to case reporting within 1 day, case investigation within 3 days, and focus investigation and public health actions within 7 days. The aim of this study was to evaluate the challenges in and lessons learned during the implementation of the 1-3-7 strategy in China so far.

**Methods:**

This qualitative study was conducted in two provinces in China: Gansu province (northwestern China) and Jiangsu province (southeastern China) in 2014. Key informant interviews (*n* = 6) and in-depth interviews (*n* = 36) about the implementation aspects of the 1-3-7 strategy were conducted with malaria experts, health staff, laboratory practitioners, and village doctors at the provincial, city, county, township, and village levels.

**Results:**

Broad themes related to the challenges in and lessons learned during the implementation of the 1-3-7 strategy were identified according to: case reporting within 1 day, case investigation within 3 days, focus investigation within 7 days, and the overall strategy. The major challenges outlined were related to respecting the timeline of surveillance procedures, the absence of or difficulties in following guidelines on conducting focus investigations, diagnostics, and the increasing number of returning migrant workers from malaria-endemic countries. Important lessons learned revolve around the importance of continuous capacity building, supervision and motivation, quality control, information technology support, applied research, governmental commitment, and intersectoral collaboration.

**Conclusions:**

Surveillance is a key intervention in malaria elimination programs. The Chinese 1-3-7 strategy has already proven to be successful but still needs to be improved. In particular, dealing appropriately with imported malaria cases through the screening of migrant workers from malaria-endemic countries is essential for achieving and sustaining malaria elimination in China. China has perfect preconditions for successful malaria elimination provided political commitment and financial investment are guaranteed. The 1-3-7 strategy may also be considered as a model for other countries.

**Electronic supplementary material:**

The online version of this article (doi:10.1186/s40249-016-0188-8) contains supplementary material, which is available to authorized users.

## Multilingual abstract

Please see Additional file 1 for translations of the abstract into the five official working languages of the United Nations.

## Background

Malaria remains the most important parasitic disease globally, with some three billion people in 97 endemic countries at risk [1]. According to estimates of the World Health Organization (WHO), malaria caused 214 000 000 disease episodes and 438 000 deaths in 2015 [1]. The overall goal of the international community is to eliminate malaria country by country until, finally, malaria eradication is achieved [2].

In 2010, China initiated its National Malaria Elimination Program (NMEP) with the goal of eliminating malaria by 2020 [3]. Continuous progress has been made through the implementation of the NMEP. Indigenous malaria was limited to 41 counties in five provinces and 12 counties in three provinces in 2012 and 2013, respectively [4, 5]. However, almost all Chinese counties have reported imported malaria cases in recent years, with these mainly being migrant workers returning from malaria-endemic countries [6, 7]. As a consequence, imported malaria cases now account for over 90 % of all malaria cases in China [8].

Well-functioning surveillance systems are of utmost importance for the successful elimination of an infectious disease [9–11]. Pockets of malaria endemicity and new outbreaks need to be rapidly identified to prevent recurrences [12]. Reactive case detection (RACD), which includes screening of communities for malaria followed by immediate treatment of positive cases, usually coupled with vector control interventions and health education, has been widely used in countries which are in malaria elimination phases [13–16]. China’s 1-3-7 surveillance and response strategy is considered an innovative combination and modification of the key components of malaria surveillance and response activities in the elimination stage. I involves reporting suspected and confirmed cases within 1 day, investigation of specific cases within 3 days, and focus investigation followed by targeted control measures (indoor residual spraying of insecticides, IRS; RACD; health education) within 7 days [17, 18]. While there have been continuous improvements in the timeliness of focus investigations and response since the beginning of the 1-3-7 strategy, the proportion of cases for which this occurs within 7 days was only around 50 % as of 2013 [17, 18].

The aim of this study was to explore the remaining operational challenges in and lessons learned during the implementation 1-3-7 strategy in order to enable further improvements.

## Methods

### Study setting

**Fig. 1:**
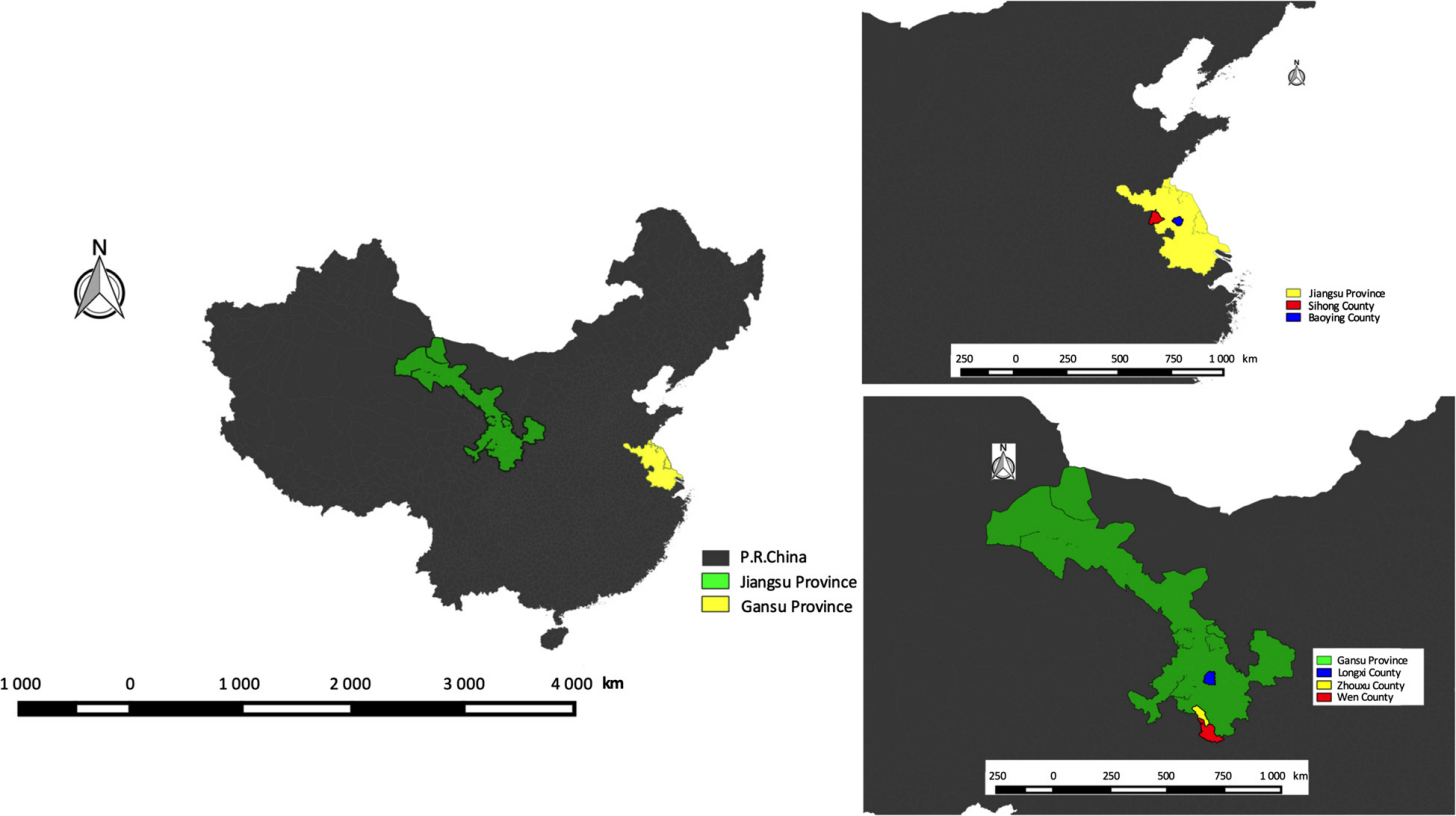
Study sites (provinces and counties) in China

**Fig. 2:**
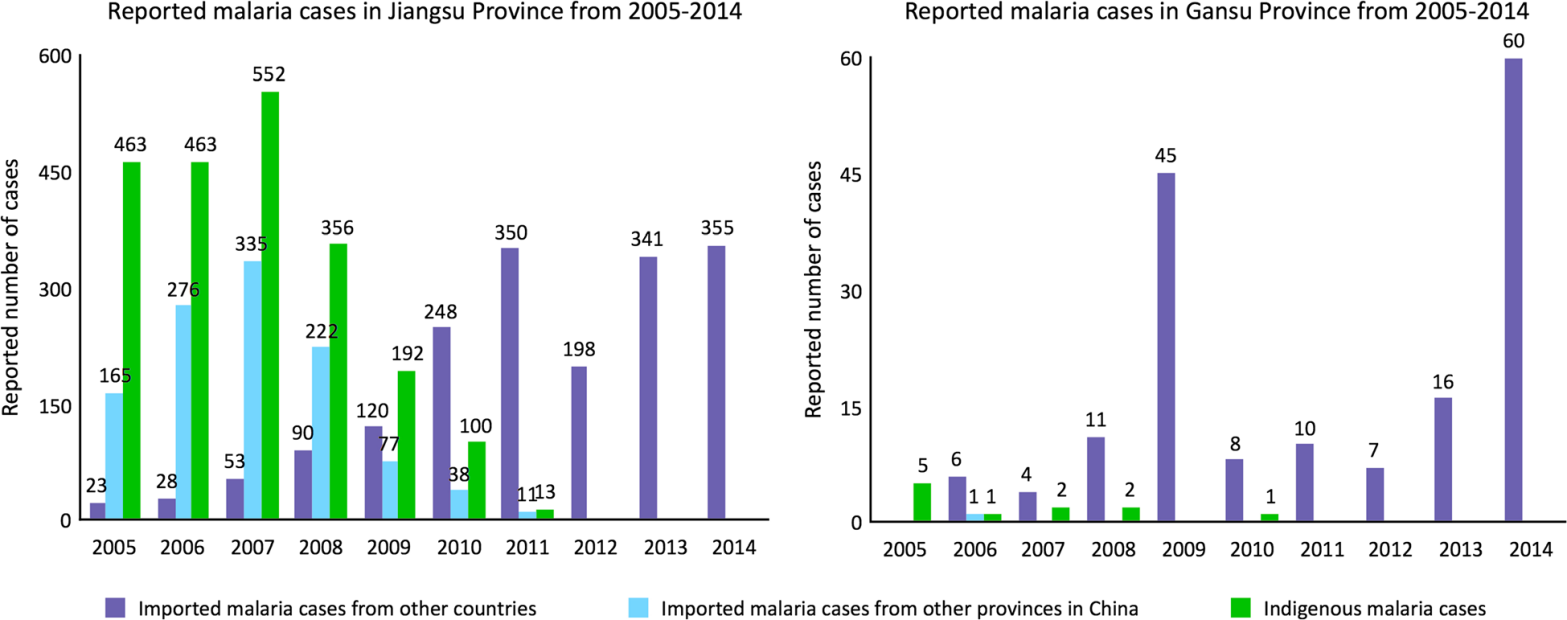
Reported malaria cases in the study provinces from 2005 to 2014

The study was conducted in two historically malaria-endemic provinces: Gansu province (northwestern China) and Jiangsu province (southeastern China) (see Fig. 1). Gansu has not reported indigenous malaria cases since 2011 and reported a total of 60 imported malaria cases in 2014. Jiangsu has not reported indigenous malaria cases since 2012 but reports high numbers of imported malaria cases (see Figs. 1 and 2) [19, 20].

### Participants and sampling strategy

**Fig. 3:**
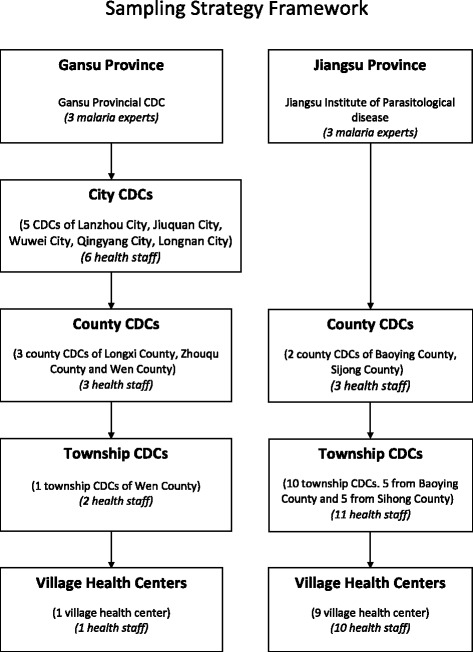
Sampling framework

The sampling procedure is shown in Fig. 3. In Gansu, five city Centers for Disease Control and Prevention (CDCs) were selected for convenience as they host the public health institutions relevant for this research. In addition, three and two county CDCs were selected according to their malaria situation in Gansu and Jiangsu, respectively (see Fig. 1). Finally, a number of township CDCs and village clinics were purposely selected for the study, mainly in Jiangsu. The selection was based on epidemiological considerations, such as malaria incidence, prevalence and distribution of vectors, etc., and specific information of local staff, such as the information for the study sites sampling and data collection.

The study participants included two groups: (1) Chinese malaria experts and policymakers and (2) health workers/epidemiologists involved in routine malaria surveillance at different levels of the health system. Intensity sampling (using information-rich cases) enhanced by snowball sampling was used [21]. Individuals who had participated in malaria case reporting, case epidemiological investigations, or specific control measures related to surveillance data over the last 3 years were considered information-rich cases.

### Data collection

Two semi-structured questionnaires were designed: one for key informant interviews with malaria experts and policymakers (see Additional file 2) and the other for in-depth interviews with other health workers (see Additional file 3). Interviews were recorded on tape. A total of 42 respondents were interviewed, ranging from malaria experts at provincial CDCs to staff at village clinics.

Before the interviews took place, one of the researchers (GL) explained the purpose and background of the study to the participants. During the interviews, the participants were encouraged to report their experiences with the malaria surveillance procedures of the 1-3-7 strategy. All participants were asked for their written informed consent before the interview.

### Data management and analysis

Interviews were transcribed into Chinese by GL. During this process, initial thoughts and ideas were noted down. This is considered an essential stage of qualitative analysis [22]. The transcribed data were then listened to, read, and re-read several times to ensure the accuracy of the transcription. This process of ‘repeated reading’ and ‘repeated listening of the recordings’ allows the researcher(s) to fully immerse themselves in the data and achieve closeness with the data [23].

The following coding process identified the features of the data, based on the original Chinese transcripts [24]. The coding process was conducted by two authors (GL and YL), followed by cross checking of the data by one author (GL).

Data analysis was conducted by applying a mix of deductive coding (based on the questionnaires) and inductive coding (to ensure no codes were missed) by the software MAXQDA 12.1.1. Themes were then identified and developed. The themes were related to both the challenges in and lessons learned during the implementation of each stage of the 1-3-7 strategy, including case reporting within 1 day, case investigation within 3 days, and focus investigation within 7 days. Overall challenges and lessons learned about the entire strategy were also identified. Coding, development, and refinement of the themes were done by two authors (GL and YL).

## Results

Table 1 summarizes the demographic characteristics and professional affiliations of the respondents. The majority of the respondents (69 %) had previously either reported malaria cases, or participated in the investigation of malaria cases or in focus investigation.

**Table 1:** Demographic characteristics of participants

	Participants (*N* = 42)	
Age	*N*	(%)
20–29	6	14.3
30–39	15	35.7
40–49	14	33.3
≥ 50	7	16.7
Gender		
Male	32	76.2
Female	10	23.8
Working positions		
Provincial level	6	14.3
City level	6	14.3
County level	6	14.3
Township level	13	31
Village level	11	26.2
Experiences with 1-3-7 approach		
Case reporting	29	69
Case investigation	29	69
Focus investigation	29	69

Table 2 shows the findings pertaining to the four major categories (case reporting within 1 day, case investigation within 3 days, focus investigation within 7 days, and overall strategy). Additional file 4 lists the selected supporting quotes about the challenges and lessons for each stage of the 1-3-7 strategy and the overall strategy.

**Table 2:** Themes of challenges and lessons learned of Chinese malaria 1-3-7 surveillance strategy

	Challenges	Lessons learned
Case reporting within 1 day	• Inadequate surveillance capacity of primary health staff • Delay of presence of malaria patients in clinics • Inadequate diagnostic tools • Village clinics are not covered under the reporting system	• Continues capacity building at primary health clinics • A well-functioning surveillance infrastructure • Technical flexibility of suspected cases reporting
Case investigation within 3 days	• Respecting the 3 days timeline • Difficulties of species identification • Subjective and technical aspects of case classification • Limited quality control of case investigation	• The importance of information technology (IT) support • New research findings
Foci Investigation within 7 days	• Complexity and difficulties during evaluation of local transmission risk • Respecting the 7 days timeline • IRS and RACD in potential active/active foci • Acceptance of actions by population • Limited quality control of focus investigation	• Logistical aspects • Community acceptability • Methods of quality control • Active screening of migrant workers and their peers upon return to China
Overall aspects of the 1-3-7 surveillance strategy	• Declining motivation of primary health staff • Increasing returning migrant workers from malaria endemic countries • The complexity to establish multi-sectorial collaboration	• High government commitment • Aspects on better targeting and management of returning migrant workers • Financial incentives • Health campaigns, education and training programs

### Case reporting within one day

#### Challenges

Four main challenges were identified. (1) The capacity for rapid case reporting is threatened by limited diagnostic skills, shortage of primary healthcare staff, and decreasing vigilance of malaria cases in case reporting within 1 day. In China, the most widely used diagnostic tool is microscopy. As fewer chances to contact with malaria cases, practitioners’ skills in performing and reading blood smears decrease. Moreover, Health workers are lack of high clinical suspicion of malaria over time. (2) The delayed presentation of malaria patients to clinics is mostly attributed to a lack of malaria knowledge and about early suspicion. Moreover, foremen (staff in charge of labors working abroad) may tell migrant workers to only take drugs at home when they are sick after coming back to China from malaria-endemic countries. In this case, the quality of drugs and compliance to complete treatment are rarely guaranteed. (3) Rapid diagnostic tests (RDTs) are thought to be easier to operate compared to microscopy, especially at primary healthcare centers, but they are not as readily available. (4) Village clinics are still not sufficiently equipped to fully participate in the surveillance system. The Chinese malaria web-based reporting system begins at the township level.

#### Lessons learned

Three main lessons were learned. (1) Continuous capacity building is important for early case detection and reporting, including performing blood smear tests from the township level upwards, training of health staff, and provision of well-equipped microscopes. (2) A well-established web-based real-time reporting system guarantees, to a large degree, the reporting of malaria cases within 1 day. The real-time reporting system is available from the township level up to the national level. Third, false reporting of cases (e.g. those which are not confirmed) within 1 day should not be viewed as a problem, as those cases can easily be deleted afterwards and no one would be blamed.

### Case investigation within three days

#### Challenges

Four main challenges were noted. (1) The complexity of the procedures (e.g. transport difficulties, limited working time, individual aspects) may lead to a delay of case confirmation or even to loss of patients during follow-up. (2) In terms of species identification, old microscopes coupled with health workers’ very limited experience in identifying malaria parasites were pinpointed as problems. Moreover, the diagnosis of rare parasites such as *Plasmodium ovale* was considered problematic for inexperienced laboratory staff. (3) Classifying cases (as indigenous or imported) is difficult because patients’ travel histories are incomplete. A patient may come from Singapore, but may have been in Sub-Saharan Africa (SSA) the week before. This is even more complicated for vivax malaria cases, where relapses may occur a long time after an individual leaves an endemic region. Additional challenges regarding case classification are technical. In practice, modern molecular biology techniques are rarely used to determine the likely origin of the malaria case and are thus not suited to case investigations. Moreover, it is considered to be difficult to discriminate malaria species strains of neighboring countries. (4) Quality control of case investigation was considered a problem. It appears rather difficult to judge how well, in reality, the epidemiological investigation has been conducted and how accurately health workers have classified cases.

#### Lessons learned

Two lessons were mentioned. (1) The importance of information technology was noted, especially in the case confirmation stage. The increasing availability of cheap smartphones enables rapid communication relating to surveillance data, including sending of photographs through microscope lenses for use in telemedicine. This can also be helpful for speeding up malaria confirmation procedures. (2) New research findings, such as loop-mediated isothermal amplification (LAMP), were considered to be helpful malaria diagnostic tools for the detection of low parasitemia. Moreover, advanced molecular biology technologies will be helpful for characterizing the origin of parasite strains for case investigations.

### Focus investigation within seven days

#### Challenges

Five main aspects were identified. (1) The evaluation of local transmission risk should be the first step in guiding proper action. Various epidemiological factors (e.g. vector presence, malaria species) have to be considered, which needs professional judgment. For example, if it is known that *Anopheles sinensis* is endemic in an area, it can be deduced that *P. falciparum* transmission is rather unlikely. In addition, the opinions and approaches of the local health staff often differ from judgments. (2) Difficulties with respecting the 7-day timeline were reported more frequently by health staff working in remote areas as they had to deal with transportation problems. Moreover, the mobility of patients (e.g. seeing doctors in hospitals in big cities or moving to a new address) was mentioned as a major challenge. (3) Vector control conducted using IRS faces a number of operational challenges, such as uncertainty pertaining to the radius of spraying. According to respondents, the radius ranged from 50 to 200 m. For RACD, a lack of standard operational procedures (SOPs) is one of the main challenges. (4) Acceptance of actions (IRS or RACD) by the population is another challenge expressed commonly by staff of primary healthcare units. (5) The quality of the implementation of focus investigations appears to be primarily hindered by a lack of SOPs.

#### Lessons learned

Four main lessons were identified. (1) To improve the logistics of surveillance activities, better communication is needed. For example, calling and making appointments with families in advance for focus investigation is important in order to avoid absences. Moreover, better availability of good transport (e.g. cars) has been mentioned as as important for assuring rapid investigations. (2) Important ethical aspects need to be considered. Firstly, there has to be community acceptance of the measures taken, which will depend on the activities being well explained to families and their neighbors, and on protecting the property and privacy of the patients and their families. It is furthermore not advisable for malaria staff to show up in white coats, as this will lead to suspicion that some dangerous disease is involved. White coats or working uniforms should only be used when spraying the houses and only after a full explanation of procedures. In any case, it is good to have the village doctors involved in the activities. (3) Lessons learned regarding quality control were on the importance of using different methods for documenting the work (e.g. taking pictures) and establishing clear responsibilities within the different administrative levels for provision and distribution of insecticides and drugs. (4) Active screening of migrant workers and their peers upon return to China from counties or villages where imported malaria cases have been reported was identified as being important from the countries or villages where imported malaria cases occurred. Furthermore, screening the workers’ social networks—potentially through information provided by export laborer companies—should facilitate the detection of potential cases.

### Aspects of the overall 1-3-7 surveillance and response strategy

#### Challenges

Three main challenges were identified. (1) The respondents observed that there is a declining motivation for case detection, and for conducting case investigations or focus investigations among staff working on the periphery of the health system. This appears to be related to heavy workloads, salary dissatisfaction, and largely no acknowledgement of the work. It also has been attributed to a number of other factors: job descriptions, which do not explicitly state that epidemiological work on malaria; doubts regarding the efficacy of IRS; and lack of financial incentives. (2) Increasing numbers of returning migrant workers from malaria endemic-countries was identified as a major challenge for the national surveillance program. It is often difficult to follow migrant workers up after they return to China due to these workers living and working at different addresses and corresponding difficulties regarding collaboration with respective authorities. The usual delay of presentation at regular health facilities, which may be due to reluctance to talk about their travel history because they are afraid of discrimination, is also worrisome. All these aspects can lead both to late reporting and excess mortality. (3) The complexity of establishing functioning multisectoral collaboration (with other ministries, e.g. exit-entry inspection and quarantine bureaus, export laborer companies, private health sector, etc.) is another major challenge. This concerns, in particular, collaboration with private sectors.

#### Lessons learned

Five lessons were identified. (1) The NMEP, including the 1-3-7 strategy, is well supported by the Chinese government. The Ministry of Health (MOH) reports on the 1-3-7 surveillance completion rate of every province on a monthly basis, which leads to transparency and motivation. Moreover, malaria surveillance and case reporting are strongly regulated by law in China. (2) The respondents indicated that there should be better targeting and management of returning migrant workers. The importance of screening workers and their peers upon return to China was mentioned, which also suggests the relevance of specific health education campaigns. Taking into account the knowledge of the village doctors in a timely manner and locations of these high-risk populations, as well as their capacity for active surveillance during regular house visits was also identified as being important. Finally, a better exchange of data within the CDC network and informal collaboration with export laborer companies are recommended. (3) Respondents clearly emphasized the importance of initiatives for increasing the motivation of health staff for all aspects of malaria surveillance by appropriate incentives (e.g. money). (4) The impact of educating the population on malaria, for example on World Malaria Day, was frequently mentioned. Health authorities organize health education campaigns including on malaria for migrant workers before they go abroad, as well as for staff of export laborer companies. Finally, training and retraining of health staff has been mentioned repeatedly as being important in keeping vigilance, and as contributing to a better understanding of the epidemiology of malaria and being able to more accurately judge the origin of the cases.

## Discussion

The 1-3-7 surveillance and response strategy is considered to be an innovative intervention for achieving the malaria elimination goal in China by 2020 [25]. To our knowledge, this study is the first to provide a comprehensive analysis of both the challenges in and lessons learned from malaria surveillance and response in a country that is in the malaria elimination phase [26]. The results generated by this study are expected to provide information not only to China for further improvement and sustainability of its NMEP, but also to benefit other countries in the phase of malaria pre-elimination or elimination to improve their programs by the adoption or adaption of the 1-3-7 strategy.

In general, the respondents of the study expressed positive perceptions regarding the 1-3-7 surveillance and response strategy. This was most notably related to the well-functioning web-based disease reporting system and the well-established county-township-village interactions, which provide the opportunity to closely examine and react to each individual malaria case. An early and rapid detection of imported malaria cases from the mobile population of migrant workers and well-implemented epidemiological investigations followed by appropriate interventions to prevent local transmission were identified as the biggest challenges faced by the strategy.

### Case reporting within one day

Rapid reporting is crucial in any malaria elimination program [27]. In the recent past, China used to have problems with the completeness of malaria case reporting, with less than 10 % of cases being reported [28]. The reporting system was largely strengthened thereafter, especially after the outbreak of SARS, through a mix of measures [25, 29]. Reporting malaria cases is now well guaranteed by the real-time reporting system. Moreover, China has defined malaria as a notifiable disease alongside other countries in the phase of malaria elimination, which greatly supports surveillance activities [30–32]. Major challenges to case reporting within 1 day were the decreasing knowledge and corresponding vigilance of health staff and the population regarding malaria. For example, returned migrant workers with malaria who are treated with antimalarial drugs by the foreman leads to a delay of these individuals in presenting at hospitals/clinics, or even to underreporting. Other important challenges are technical, in particular the detection of cases with low parasitemia and asymptomatic cases. This is a problem in many countries that are trying to eliminate malaria, and it is currently being hotly debated how big the problem of asymptomatic cases is and if new tools such as LAMP could be an alternative or an addition to microscopy and RDTs [33–36].

### Case investigation within three days

At the county and township levels, the main diagnostic tool is microscopy. Malaria cases are usually confirmed at a higher level through more professional microscopic examinations or even polymerase chain reaction. The challenges associated with these diagnostic procedures (e.g. availability of skilled microscopists, maintenance of technical equipment, systematic quality control) are supported by the respondents’ statements and are in accordance with findings from other malaria-endemic countries [6, 37, 38]. Another challenge is the identification of uncommon malaria species such as *P. ovale* by all microscopists [25, 39]. There are obvious differences in malaria diagnostic capacities in different provinces in China [40, 41]. This is supported by this study’s comparison of townships in Jiangsu and Gansu. Even in Yunnan province, a southern area of China bordering Myanmar, only 77 out of 131 (59 %) health facilities were able to carry out quality malaria microscopy when surveyed in 2010 [40]. In the central Guizhou province, out of 67 confirmed malaria cases, 27 (40 %) were initially diagnosed falsely negative in 2011 [42]. A survey performed in Cheng’an county (Hebei province) in 2010—a pilot site for malaria elimination—showed that there was a huge shortage of staff that was able to diagnose malaria [43]. It is thus relevant that a number of participants in this study mentioned the potential of modern communication technology for malaria diagnosis (e.g. sending photos taken through the lens of a microscope using a smartphone to a professional and getting a quick response). With the help of such simple forms of telemedicine, health staff working in remote areas could significantly decrease the time gap for case confirmation. Another important aspect of case investigation within 3 days is to classify reported malaria cases into imported or indigenous cases [17, 18, 25]. Currently, classification largely depends on subjective information from the patients together with the health workers’ professional judgment. Moreover, in the case of *P. vivax* malaria, it is particularly difficult to discriminate between relapse and reinfection [9, 44]. In this regard, findings from this study also confirm the importance of quality control and epidemiological knowledge during the phase of malaria elimination [45].

### Focus investigation within seven days

Before taking specific actions to stop further transmission in a malaria focus, one important procedure is evaluating transmission risk [2, 17]. The evaluation should be based on the information gained in the previous case investigation combined with an analysis of local epidemiological aspects. The result of the evaluation will guide proper actions by classifying the malaria foci into active, pseudo, or inactive [17]. However, in practice, it is noticed that this step is either missing or done in a non-standardized manner. Moreover, the uncertainty of further transmission risk interferes with rational policy decisions. For example, in Jiangsu, a focus on an imported *P. falciparum* malaria case in areas of *An. sinensis* only requires demographic RACD, while a focus on an imported *P. vivax* malaria case requires geographic RACD and IRS (due to the existence of a susceptible vector) [17]. In practice, some health staff would rather conduct demographic RACD and IRS, even if they think it is a pseudo focus based on the principle of ‘doing more is better than missing something’. However, other health workers argued that they would try to avoid doing IRS as it may cause panic in the community. In order to cope with these operational challenges, SOPs are needed for different epidemiological scenarios.

These challenges are exemplified by missing guidelines for RACD. The WHO suggests covering a large population, given that the flight range of the *Anopheles* mosquito is typically 1–2 km [46]. In practice, and as also found in this study, decisions regarding an appropriate intervention area have been rather arbitrary [47]. Standard operational procedures implemented by countries aiming for elimination (Sri Lanka in 2009, Swaziland in 2010) defined a 1-km radius for RACD [13, 48]. However, a 1-km radius has proven to be logistically challenging and appears unsustainable in Swaziland [13]. A malaria elimination feasibility assessment undertaken in Zanzibar identified the need for active case detection among approximately 100 neighboring households around each identified case in order to prevent the reemergence of malaria [49]. Obviously, the feasibility of such complex interventions also depends, to a large degree, on their acceptance by the population [50]. Finally, without well-established SOPs, the quality of focus investigations is hard to judge.

### Aspects of the overall 1-3-7 strategy

The strong commitment to eliminate malaria by the Chinese government greatly supports the functioning of the 1-3-7 strategy. This strong commitment involves both sufficient domestic financial investment as well as sustained supervision at all levels of the public health system.

One big challenge of the entire strategy concerns imported malaria. In China, imported malaria cases are mainly migrant workers coming back from SSA [51]. Active surveillance activities (such as screening the peers of index cases) aimed at at-risk populations have been considered to be promising for early malaria case detection, particularly in counties with high numbers of imported malaria cases. The success of this strategy has been demonstrated in 2013 in four Chinese provinces, where the screening of 4358 peers from 167 index cases led to the detection of an additional 737 imported malaria cases [52]. This example also highlights the importance of improved community participation and increased involvement of village doctors in malaria case detection, which was also frequently mentioned by the participants of this study.

When the Chinese MOH, along with other 13 ministries and commissions, issued the NMEP, the importance for intersectoral collaboration was emphasized [52]. However, this is not an easy subject as there are conflicts of interest to be considered, such as companies being worried about the acceptance of further enrollment of migrant workers for jobs in malaria-endemic countries. Good communication about the risk of malaria and the purpose of screening returning workers are necessary to establish functioning collaborations with local companies and various governmental departments. The participants of this study identified some promising communication strategies. For example, health workers have told companies that detailed information on migrants is required (e.g. numbers, destinations, and home places) to prepare for sufficient antimalarial drug supplies in Chinese counties.

### Strengths and limitations of the study

A strength of this study is the fact that all interviews were conducted by one researcher who does not work for a Chinese authority or organization, and this may have decreased the pressure on the respondents to give answers perceived as being in line with current policies. This is also, to our knowledge, the first large-scale qualitative study investigating China’s 1-3-7 strategy. A limitation is that the study areas are not representative of the whole of China.

## Conclusions

The main goal of NMEPs is to achieve malaria elimination and to sustain this. The 1-3-7 strategy has already proven to be valuable for the NMEP in China, but it still needs to be improved. Dealing appropriately with the specific aspects of imported malaria cases (i.e., imported through workers returning from malaria-endemic countries, as well as by tourists and other migrants) will remain a major challenge for achieving and sustaining malaria elimination in China. However, given the impressive socioeconomic development and ongoing improvements in the health sector, the country has the perfect preconditions for successful elimination provided continuous political commitment and financial investment are guaranteed. The 1-3-7 strategy may also be considered a model for other countries, which may adapt it according to the lessons learned in China and their local circumstances.

## Additional files

Additional file 1: Multilingual abstracts in the five official working languages of the United Nations. (PDF 805 kb)
Additional file 2: Questionnaire for in-depth interviews. (PDF 150 kb)
Additional file 3: Questionnaire for key informant interviews. (PDF 113 kb)
Additional file 4: Table S1. Selected supporting quotes about challenges and lessons learned in case reporting within 1 day. **Table S2.** Selected supporting quotes about challenges and lessons learned in case investigation within 3 days. **Table S3.** Selected supporting quotes about challenges and lessons learned in focus investigation within 7 days. **Table S4.** Selected supporting quotes about challenges and lessons learned from the entire 1-3-7 strategy. (PDF 92 kb)

## Abbreviations

CDC: Center for Disease Control and Prevention
IRS: indoor residual spraying of insecticides
LAMP: loop-mediated isothermal amplification
MOH: Ministry of Health
NMEP: National Malaria Elimination Program
RACD: reactive case detection
RDT: rapid diagnostic test
SOP: standard operational procedure
SSA: Sub-Saharan Africa
WHO: World Health Organization
