# Supplementary material for: Challenges in and lessons learned during the implementation of the 1-3-7 malaria surveillance and response strategy in China: a qualitative study
Source: Infect Dis Poverty. 2016 Oct 5;5:94. doi: 10.1186/s40249-016-0188-8 (PMC5050603; doi:10.1186/s40249-016-0188-8)
Supplement: Additional file 1: — Multilingual abstracts in the five official working languages of the United Nations. (PDF 805 kb) [file 40249_2016_188_MOESM1_ESM.pdf]

Translation of the abstract into the six official working languages of the United Nations

التحديات والدروس المكتسبة من تطبيق ترصد الملاريا 1-3-7 واستراتيجية الاستجابة في الصين: دراسة نوعية

جوان - يو لو، ياو-باو ليو، كلوديا بييرسمان، يو فينغ، جون كاو، أولاف مولير

ملخص
خلفية: حققت الصين تطورا تقدما كبيرا في مجال مكافحة الملاريا على مدى القرن الماضي، وهي الآن تهدف إلى القضاء على الملاريا بحلول العام 2020. وفي عام 2012، أطلقت الصين استراتيجية ترصد الملاريا والاستجابة لها رقم 1-3-7. توثق الاستراتيجية حالات الملاريا المبلغ عنها خلال يوم واحد، والتحقيق فيها خلال ثلاثة أيام، وإجراءات التحقيقات المركزة والصحة العامة المتخذة خلال سبعة أيام. وتهدف هذه الدراسة إلى تقييم التحديات التي تم مواجهتها والدروس المكتسبة من تنفيذ الاستراتيجية 1-3-7 في الصين حتى اللحظة.
الأساليب: أجريت هذه الدراسة النوعية في مقاطعتين في الصين: مقاطعة قانسو (شمال غرب الصين) ومقاطعة جيانغسو (جنوب شرق الصين) في عام 2014. تم عقد مقابلات مع عدد من المصادر الرئيسية للمعلومات (بعدد 6)، وعدد من المقابلات المتعمقة (بعدد 36) مع خبراء مختصين في مرض الملاريا، والعاملين في مجال الصحة، والعاملين في المختبرات، وأطباء القرى في المقاطعات والمدن والبلدات والقرى، وذلك حول الجوانب المرتبطة بتنفيذ الاستراتيجية 1-3-7.
النتائج: تم تحديد الموضوعات العامة المتعلقة بالتحديات والدروس المستفادة أثناء تنفيذ الاستراتيجية 1-3-7 وفقاً لـ : حالات الملاريا المبلغ عنها خلال يوم واحد، والتحقيقات المجرية خلال ثلاثة أيام، وإجراءات التحقيقات المركزة والصحة العامة المتخذة خلال سبعة أيام، والاستراتيجية ككل. تتعلق التحديات الرئيسية المذكورة بالالتزام بالجدول الزمني لإجراءات الترصد، وغياب أو صعوبات اتباع التوجيهات المرتبطة بإجراء التحقيقات المركزة، والجوانب التشخيصية، والعدد المتزايد من العمال المهاجرين القادمين من البلدان الموبوءة بالملاريا. تتركز الدروس المهمة المكتسبة حول أهمية عملية بناء القدرات المستمر، والإشراف والتحفيز، ورقابة الجودة، ودعم تكنولوجيا المعلومات، والبحوث التطبيقية، والالتزام الحكومي، والتعاون بين مختلف القطاعات.
الاستنتاجات: تعد عملية الترصد عامل الانقاذ الأساسي في برامج التخلص من الملاريا. وقد أثبتت الاستراتيجية 1-3-7 الصينية نجاحها بالفعل ولكنها لا تزال بحاجة إلى بعض التحسينات. وتحديدا فيما يتعلق بالتعامل الملائم مع حالات الملاريا من الخارج وذلك من خلال الفحوصات الأولية للعمال المهاجرين من البلدان الموبوءة بالملاريا والذي يعد ضروريا لتحقيق التخلص من الملاريا واستدامته في الصين. تطبق الصين شروط مسبقة مثالية لنجاح عملية التخلص من الملاريا والمرهونة بضمان الالتزام السياسي والاستثمارات المالية. ويمكن أيضا النظر في الاستراتيجية 1-3-7 كنموذج يحتذى به في دول أخرى.

Translated from English version into Arabic by Nada Khwairah, through

TRANSLATORS
WITHOUT BORDERS

中国消除疟疾“1-3-7”策略实施过程的中挑战和经验的定性研究

卢光玉，刘耀宝，Claudia Beiersmann，冯宇，曹俊，Olaf Müller

摘要
引言：中国的疟疾防治在过去的一个世纪取得了巨大的成就，目前的目标是在 2020 年全国实现消除疟疾。在 2012 年，中国开展了消除疟疾监测与响应的“1-3-7”策略，即所有疟疾病例在诊断后 1 天内通过疫情报告系统上报，在 3 天内完成病例个案调查和病例核实，在 7 天内完成疫点调查与处置。本研究的目的是评估中国“1-3-7”策略实施至今所面临的挑战和积累的经验。
方法：本定性研究于 2014 年在中国西北部的甘肃省和东南部江的苏省开展。对实施“1-3-7”策略的省、市、县、乡、村等各级医疗卫生机构的工作人员进行访谈，访谈对象包括疟疾专家、疾控中心专业人员、实验人员和乡村医生，其中对 6 人进行了关键知情人访谈，对 36 人进行了深度访谈。
结果：研究结果包括挑战和经验两部分，并按照“1-3-7”策略的实施环节（1 天疫情上报，3 天病例个案调查和 7 天疫点调查）进行分类整理。主要的挑战包括：监测措施完成的及时性、缺乏疫点处置技术指南和实施存在困难、诊断方面的困难、以及从疟疾流行国家返乡的务工人员持续增加。主要的经验包括：监测能力建设、监督和激励措施、质量控制、信息技术的支持、应用研究的开展、以及政府的承诺和跨部门合作。
结论：监测是消除疟疾工作中一个关键的干预措施。中国的“1-3-7”策略已经证明是成功的，但仍需要进一步改进。特别是通过筛查从疟疾流行国返乡的出国务工人员来应对输入性疟疾，从而实现和维持中国疟疾消除目标。中国有较好的政治承诺和资金投入，这为消除疟疾目标的实现奠定了坚实的基础。同时，中国的“1-3-7”策略也为其他国家提供了借鉴和参考。

Translated from English version into Chinese by Guang-Yu Lu

Difficultés et enseignements du déploiement de la stratégie de surveillance et de réponse au paludisme «1-3-7» en Chine: étude qualitative

Guan-Yu Lu, Yao-Bao Liu, Claudia Beiersmann, Yu Feng, Jun Cao, Olaf Müller

Résumé

Contexte: La Chine a fait d’importants progrès dans la lutte contre le paludisme au cours du siècle écoulé et vise aujourd’hui à éliminer la maladie d’ici 2020. Le pays a lancé sa stratégie de surveillance «1-3-7» en 2012. Il s’agit de parvenir à un signalement des cas sous un jour, leur investigation sous trois jours et des investigations des foyers et actions de santé publique sous sept jours. Le but de notre étude était d’évaluer les difficultés du déploiement de cette stratégie et les enseignements qui peuvent d’ores et déjà en être tirés.
Méthodes: Cette étude qualitative a été menée en 2014 dans deux provinces chinoises: la province du Gansu (nord-ouest) et celle du Jiangsu (sud-est). Des entretiens avec des informateurs importants (*n*=6) et des entretiens approfondis (*n*=36) ont été menés à propos du déploiement de la stratégie «1-3-7» avec des experts du paludisme, des personnels de santé, des biologistes de laboratoire et des médecins de village au niveau des province, des grandes villes, des circonscriptions, des villes et des villages.
Résultats: Les grands thèmes liés aux difficultés et enseignements du déploiement de la stratégie «1-3-7» ont été identifiés en relation avec la notification des cas sous un jour, leur investigation sous trois jour, l’investigation des foyers sous sept jours et avec la stratégie dans son ensemble. Les principales difficultés mises en lumière concernaient le respect des délais des procédures de surveillance, l’absence de directives sur les investigations des foyers ou la difficulté à suivre celles qui existent, les questions de diagnostic et le nombre croissant de travailleurs migrants qui reviennent de pays où le paludisme est endémique. Les enseignements tirés concernent l’importance de la création continue de capacités, de la supervision et de la motivation, du contrôle de qualité, de l’assistance par les technologies de l’information, de la recherche appliquée, de l’engagement des pouvoirs publics et de la collaboration intersectorielle.
Conclusions: Dans les programmes d’élimination du paludisme, la surveillance joue un rôle essentiel. La stratégie «1-3-7» chinoise est déjà un succès mais elle peut encore être améliorée. La gestion des cas de paludisme importés, en particulier, est indispensable pour obtenir une élimination durable du paludisme dans le pays. Elle nécessite un dépistage sur les travailleurs migrants en provenance de pays où le paludisme est endémique. La Chine a créé les conditions idéales pour réussir à éradiquer le paludisme, à condition que la volonté politique et les engagements financiers se maintiennent. En outre, la stratégie «1-3-7» peut être considérée comme un modèle pour d’autres pays.

Translated from English version into French by Suzanne Assenat, through

TRANSLATORS
WITHOUT BORDERS

Трудности и выводы, сделанные в процессе реализации "стратегии 1-3-7" по надзору и реагированию на вспышки малярии в Китае: качественное исследование

Гуан-Ю Лу, Яо-Бан Лиу, Клаудиа Байерсманн, Ю Фенг, Джун Цао, Олаф Мюллер (Guan-Yu Lu, Yao-Bao Liu, Claudia Beiersmann, Yu Feng, Jun Cao, Olaf Müller)

Отрывок
История вопроса: За последнее столетие Китай достиг значительного прогресса в деле борьбы с малярией, и к 2020 году намеревается окончательно искоренить данное заболевание. В 2012 году страна приступила к реализации "стратегии 1-3-7" надзора за малярией и реагирования на ее вспышки. Данная стратегия состоит в передаче уведомления о вспышке малярии в течение одного дня, изучении случая в течение трех дней и фокусного исследования и принятия мер в области общественного здравоохранения в течение семи дней. Цель настоящего исследования заключается в оценке трудностей и выводов, сделанных в процессе реализации "стратегии 1-3-7" в Китае на настоящий момент.
Методы: Данное качественное исследование было проведено в двух китайских провинциях: в провинции Ганьсу (на северо-западе Китая) и провинции Цзянсу (на юго-востоке Китая) в 2014 году. Были проведены основные информационные интервью (*n*=6) и глубинные интервью (*n*=36) с экспертами по малярии, работниками системы здравоохранения, работниками лабораторий и деревенскими докторами на уровне провинции, города, округа, поселка и деревни относительно аспектов внедрения "стратегии 1-3-7".
Результаты: Был рассмотрен широкий круг тем относительно трудностей и выводов, сделанных в процессе внедрения "стратегии 1-3-7", на основании: передачи уведомления о вспышке заболевания в течение одного дня, изучения случая в течение трех дней, фокусного исследования в течение семи дней и стратегии в целом. Среди основных трудностей были обозначены соблюдение графика процедур надзора, отсутствие руководящих указаний по проведению фокусных исследований или трудности в следовании им, диагностические аспекты, а также растущее количество рабочих-мигрантов, возвращающихся из стран, в которых отмечена эндемия малярии. Были сделаны важные выводы относительно значимости непрерывного наращивания потенциала, надзора и мотивации, контроля качества, поддержки информационных технологий, прикладных исследований, целенаправленной государственной политики и межотраслевого взаимодействия.
Выводы: Основной оперативной мерой в программах по искоренению малярии является эпидемиологический надзор. Китайская "стратегия 1-3-7" уже доказала свою эффективность, но еще нуждается в доработке. В частности, надлежащее обращение с завезенными случаями заболеваний малярией посредством обследования рабочих-мигрантов,

приезжающих из стран, где зафиксирована эндемия малярии, является основой для окончательного искоренения малярии в Китае. В Китае имеются идеальные предпосылки для успешного искоренения малярии, при наличии политической воли и финансовых инвестиций. "Стратегию 1-3-7" можно также рассматривать в качестве модели для других стран.

Translated from English version into Russian by tatiana_com, through

TRANSLATORS
WITHOUT BORDERS

Desafíos y lecciones que aprendimos durante la implementación de la estrategia 1-3-7 para el monitoreo y respuesta ante la malaria en China: Un estudio cualitativo

Guan-Yu Lu, Yao-Bao Liu, Claudia Beiersmann, Yu Feng, Jun Cao, Olaf Müller

Resumen

Antecedentes: En este último siglo China ha logrado progresar significativamente en el control de la malaria y ahora tiene como objetivo eliminar la enfermedad para el año 2020. En el año 2012 el país lanzó la estrategia 1-3-7 para el monitoreo y respuesta ante la malaria. La estrategia consiste en el reporte de casos dentro de un día, la investigación de casos dentro de los tres días y la investigación dirigida y acciones de salud pública dentro de los siete días. El objetivo del presente estudio fue evaluar los desafíos y las lecciones aprendidas durante la implementación de la estrategia 1-3-7 en China hasta la fecha.

Métodos: Este estudio cualitativo se condujo en dos provincias de China: La provincia de Gansu (en el noroeste de China) y la provincia de Jiangsu (en el sureste de China) en el año 2014. Se condujeron entrevistas de actores claves (n=6) y entrevistas en profundidad (n=36) sobre los aspectos de implementación de la estrategia 1-3-7 con expertos en malaria, trabajadores de la salud, laboratoristas, y médicos a nivel provincia, municipio, condado, aldea y poblado.

Resultados: Se identificaron temas amplios relacionados con los desafíos y las lecciones aprendidas durante la implementación de la estrategia 1-3-7 de acuerdo a: reporte de casos dentro de un día, investigación de casos dentro de los tres días, investigaciones dirigidas dentro de los siete días y la estrategia en general. Los principales desafíos que se identificaron se relacionaban con el respeto de la cronología de los procedimientos de monitoreo, la ausencia de o dificultad en el seguimiento de normativas para la conducción de investigaciones dirigidas, aspectos diagnósticos, y el creciente aumento de trabajadores inmigrantes que regresan de países donde la malaria es endémica. Las lecciones importantes que se aprendieron giran en torno a la importancia de la continuidad en el desarrollo de capacidades, supervisión y motivación, control de calidad, apoyo de tecnología de la información, compromiso del gobierno y cooperación intersectorial.

Conclusiones: El monitoreo es una intervención clave en los programas de eliminación de la malaria. Ya se ha demostrado el éxito de la estrategia 1-3-7 de China pero todavía necesita mejorarse. En particular, para lograr y mantener la eliminación de la malaria en China es fundamental el manejo adecuado de los casos importados de malaria mediante un tamizaje de los trabajadores inmigrantes de países donde la malaria es endémica. China tiene las condiciones perfectas para una eliminación exitosa de la malaria siempre y cuando se garanticen el compromiso político y la inversión financiera. La estrategia 1-3-7 también puede ser considerada como modelo para otros países.

Translated from English version into Spanish by Maria Alejandra Aguada, through

TRANSLATORS
WITHOUT BORDERS
