# Supplementary material for: Challenges in and lessons learned during the implementation of the 1-3-7 malaria surveillance and response strategy in China: a qualitative study
Source: Infect Dis Poverty. 2016 Oct 5;5:94. doi: 10.1186/s40249-016-0188-8 (PMC5050603; doi:10.1186/s40249-016-0188-8)
Supplement: Additional file 2: — Questionnaire for in-depth interviews. (PDF 150 kb) [file 40249_2016_188_MOESM2_ESM.pdf]

Questionnaire for in-depth interviews

Study information to respondents

My name is Guangyu Lu, and I am a doctoral student from Institute of Public Health, Medical Faculty, University of Heidelberg, Germany. We are conducting a study as part of my doctoral project on ''*Malaria elimination in China: Challenges to national and local health information and malaria surveillance systems. Part II: challenges and experience of present malaria 1-3-7 surveillance strategy in China*''. This research is purely academic and will not be used for any business purposes or profit related issues. I want to invite you take part in and will ask you some questions on the implementation of current Chinese 1-3-7 malaria surveillance strategy.

The interview will take rough 1 hour and will be tape-recorded. All information you give will be confidential. You could withdraw at any time you want. The content of the interview will be analyzed anonymously. You could contact me at any time if you have questions.

Do you agree to participate in this study?

1 Yes ☐ 2 No ☐

Socio- demographic characteristics of the respondents

1. Name: ______________________________________
2. ID: ____
3. Date of interview: _____________________________
4. District: ____________________________________
5. Age: ____
6. Gender: ______________________________________

7: Working position of respondent: __________________

8: Duration in current position: ______________________

9: Education level: _______________________________

Introduction of local situation

1. How many years have you been working in the area of malaria? (Icebreaker question)

----

2. What is the malaria situation in in your working city/county/township/village?

----

3. Aare there any changes of malaria situation in your place in recent years?

----

4. How the malaria surveillance system runs in your county?

Understanding of the 1-3-7 malaria surveillance strategy

1. Do you know the 1-3-7 malaria surveillance strategy?

Yes☐ No☐ A little bit☐

1.1 If yes/a little bit, how did you know it?

1.2 If yes/a little bit, Could you please give an explanation of the 1-3-7?

1.3 If no, then the researcher will explain the procedures to respondents

----

2. Is this strategy already implemented in your city/county/township/village? (If so, since when? If no, why?)

Specific operational aspects of case reporting and case investigation

1. In which case will you consider reporting a malaria case?
(Prompts:
-Suspected malaria cases, then how will you suspected?)
-Labor confirmed, then by which lab method?)

----

2. By which means did you report a malaria case?
(Prompts:
-Computer?)
-Telephone?
-Paper?)

----

3. Do you ever have difficulties regarding malaria case reporting?
(Prompts:
-If yes, please tell the detail of the difficulties
-If no, please tell what facilitators helped you on case reporting)

---

4. What are the recommended malaria diagnostic tools?
(Prompts: microscopy/RDT/PCR)

---

5. What is the current using malaria diagnostic tool in your working station/place?

---

6. What do you think of this tool?
(Prompts:
-Sensitivity
-Cost
-Easy doing)

---

7. What do you think of the function of infectious disease information reporting system?
(Prompts:
-Easy doing
-Accessibility
-Reasonability of setting/items)

---

8. What do you think of the “malaria cases confirmation within 3 days”?
(Prompts: aspects of feasibility/accuracy/costing)

---

9. Have you ever experienced any difficulties regarding confirming malaria cases within 3 days?
(Prompts:
-If yes, please tell the detail of the difficulties
-If no, please tell what facilitators helped you regarding case confirmation)

---

10. What do you think of the strategy “malaria case classification within 3 days”?
(Prompt: aspects of feasibility/method/accuracy/costing/quality control)

---

11. How will you classify a malaria case (imported or indigenous)?

---

12. Do you ever have/experienced any difficulties regarding classifying the origin of malaria cases within 3 days?
(Prompts:

-If yes, please tell the detail of the difficulties
-If no, please tell what facilitators helped you regarding case classification)

----

13. If a malaria case is confirmed, and then what will you do subsequently?
(Prompts:
-Case investigation
-Evaluation of the risk of the foci
-Focus investigation actions)

----

14. Have you ever traced malaria patients among mobile population (e.g., migrant workers or flowing population)?

14.1 If yes, are there any difficulties?

14.1 If no, how did you managed it?

----

15. What do you think of completing malaria case investigation within 3 days?
(Prompts:
-Feasibility/accuracy/costing/necessity of the timeframe 3 days

----

16. Are there any difficulties in completing malaria case investigation within 3 days?
(Prompts:
-If yes, what are the some potential solutions?)

----

17. What is the definition of an imported malaria case?
(Prompts:
-Importation from endemic region of China or endemic countries?)

----

18. How do you classify an imported malaria case in your work?
(Prompts:
-Travelling history)

Operational difficulties and lessons in focus investigation

1. What is a focus investigation by your understanding?

----

2. In your opinion, what is the purpose of conducting a focus investigation?

----

3. In which conditions the focus investigation will be triggered?
Prompts:
(Prompts:
-Listen to the order/suggestions from the provincial level
-Imported malaria cases found?
-Indigenous malaria cases found?
-Active vectors?
-Cases occur during transmission seasons?)

----

4. Do you know what are active foci? If so, what is it?

----

5. Have you ever conducted/participated a focus investigation?

Yes☐ No☐

5.1 If yes---
How did you/the team decided to do this/these focus investigation(s)?

What have you done during this/these focus investigation(s)?

5.2 If no, could you please tell me the reason?
(Prompts: -No active foci
-Logistic reasons
-Limited experiences)

----

6. Is there any standard operational procedures or manuals acting as guidelines for your actions during focus investigation?

6.1 If yes, which organization or who launched/give you this guideline?

6.2 If no, then how did you organize/conduct the work?

----

7. When will you consider conducting IRS?

----

8. Regarding the IRS, is there difficulties from your experiences?

Yes ☐ No ☐

8.1 If yes, what is it?

8.2 If no, what factors facilitates you work?

8.3 Could you please describe the detailed actions reharding spraying the patients' house/room?
(Prompts:
-How to define which rooms that should be sprayed?
-To which extent should the rooms be sprayed?)

8.4 How large radius did you / will you think the neighborhood should be sprayed?
(Prompts:
-By how large radius?
-By number of the neighbors?)

8.5 How about the community acceptability of the IRS?
(Prompts:
-Any difficulties?
-Any Solutions?)

----

9. Regarding the RACD, is there any difficulties from your experiences?

Yes ☐ No ☐

9.1 If yes, what is it?

9.2 If no, what factors facilitates you work?

9.3 Could you please describe the detailed actions of the RACD?
(Prompts:
-How to define when the RACD should be done?)

9.4 How large radius did you or will you think the RACD should be done?
(Prompts:
-Meters?
-Number of the neighbors?)

9.5 How is the community acceptability of the RACD?

----

10. Have you ever observed/experienced any difficulties regarding the focus investigation?
(Prompts:
-Acceptance
-Transportation
-Logistics
-Understanding)

----

11. What do you think of the timeframe to conduct focus investigation within 7 days?
(Prompts:
-Remote areas
-Hard to follow population
-Costing
-Lack of guideline
-Lack of experience)

----

12. How many people usually need to conduct a focus investigation?
(Or how many people do you think need for conducting a focus investigation)?

----

13. Whom should/ usually in practice participate the focus investigations?

----

14. What do you think of sending health care staff to the filed for several days to conduct a focus investigations?
(Prompts:
-Enough staff?
-Costing?
-Working labor?)

----

15. What kind of transportation tools do you usually use to go to the filed to do focus investigation?
(Prompts:
-It depends on distance like car, train and bus?
-It depends on budgets?
-It depends on the road condition?)

----

16. What do you think of the current transportation support/condition for conducting focus investigation within 7 days?
(Prompts:
-Well organized
-Lacking of supply
-Traffic jams
-Budgets
-Long distance or remote areas)

----

17. Do you need to pay for the transportation when you conducted focus investigation?

----

18. How about the specific costing regarding conducting the focus investigations?
(Prompts:
-What needs to be pay?
-Who will pay for what?)

----

19. What do you think of providing awards/incentives for health care staff, who conducted a focus investigation within 7 days?

----

20. What will you do with the result of the focus investigations?

(Prompts: paper reporting/ Paper publishing/To whom report/Computer saved/Share)

----
21. What aspects you think need to be strengthened to promote the health care staffs finish the focus investigation within 7 days?
(Prompts: -Human resource and administrative
-Finical support
-Logistic support
-CDC internal and external exchange and communication
-Population movement management
-Standard operational guidance

The malaria-related working aspects of village doctors and township health care staff

1. What are your routine workings?

----
2. How have you become a village doctor or township doctors/township health care staff?

----
3. What are the requirements to work in your position (qualifications)?

----
4. How do you feel like working in your position?

----
5. Have you done any work related to malaria?

Yes ☐ No ☐
5.1 If yes, what is it?

5.2 If no, why?

----
6. What kind of patients have you most commonly seen?
(Prompts:
-Fever patients?)

----
7. Nowadays, if a fever patient comes to see you, would you consider malaria?

Yes ☐ No ☐

7.1 If yes, how could you consider malaria?
(Prompts:
-Travelling history?
-Working abroad?)

7.2 If no, why?

----
8. Do you have an idea if anybody in your village/area works abroad?

----
9. Do you have an idea if anybody in your village/area returning back if he/she works abroad?

----
10. Was there imported malaria cases occurred previously in your village/township?

Yes ☐ No ☐

10.1 If yes, how did the imported malaria case(s) have been detected?

And what have you done subsequently after detection of the imported case?

10.2 If no, how do you think about imported malaria cases?
(Prompts
-Is it a threatening?
-Is it under control?)

----
11. What do you think about malaria cases management in your work?

----
12. Have you participate in spraying or focus investigation before?

12.1 If yes, could you describing the detailed actions you did?

And have you felt any difficulties?

----

13. What do you think of detecting malaria cases?

----

14. What do you think of detection malaria cases by you from your recruiting of patients?

----

15. How was your salary?

----

16. What do you think of salary?

----

17. Are there difficulties during your work related to malaria cases detection/management?

Yes ☐ No ☐

17.1 If yes, what kind of difficulties?
(Prompts
-Lacking of working time?
-Lacking of incentives?
-Lacking of guidelines?
-Losing of priority?
-Lacking of supervision?)

17.2 If no, what are the lessons/experiences?

----

18. How did you feel about your job?

----

1. What factors you think might hamper your working?

----

2. What do you think could/need to improve to make your working better?

Communication of the 1-3-7 approach between different administrative levels

1. Have you ever asked assistances from provincial level regarding malaria case investigation?

Yes ☐ No ☐

1.1 If yes, what is it?

----

2. In which kind of situations will you consider to ask assistances from superior malaria facilities/institute?

----

3. Is there any problems regarding requesting/providing help between your working place and superior/ inferior facilities?

Yes ☐ No ☐

3.1 If yes, what kind of gaps?

(Prompts-communication/policy/conflict of interest)

3.2 If no, what facilitates the communication?

Malaria related training programs

1. How frequent do you usually get training?

----

2. What kinds of training do you get?

----

3. What do you think of getting regular training on malaria? (With malaria elimination setting and very few malaria cases)

----

4. What training will you expect to have in the future?

Thank you for your time and answers!
