# Supplementary material for: Challenges in and lessons learned during the implementation of the 1-3-7 malaria surveillance and response strategy in China: a qualitative study
Source: Infect Dis Poverty. 2016 Oct 5;5:94. doi: 10.1186/s40249-016-0188-8 (PMC5050603; doi:10.1186/s40249-016-0188-8)
Supplement: Additional file 3: — Questionnaire for key informant interviews. (PDF 113 kb) [file 40249_2016_188_MOESM3_ESM.pdf]

Questionnaire for key informant interviews

Study information to respondents

My name is Guangyu Lu, and I am a doctoral student from Institute of Public Health, Medical Faculty, and University of Heidelberg, Germany. We are conducting a study as part of my doctoral project on ''*Malaria elimination in China: Challenges to national and local health information and malaria surveillance systems. Part II: challenges and experience of present malaria 1-3-7 surveillance strategy in China*''. This research is purely academic and will not be used for any business purpose or profit related issues. I want to invite you take part in and will ask you some questions on the implementation of current 1-3-7 malaria surveillance strategy and your opinions on the malaria surveillance strategy in China

The interview will take rough 1 hour and will be tape-recorded. All information you give will be confidential. You could withdraw at any time you want. The content of the interview will be analyzed anonymously. You could contact me at any time if you have questions.

Do you agree to participate in this study?

1 Yes ☐ 2 No ☐

Socio- demographic characteristics of the respondents

1. Name: ____________________________________
2. ID: ____
3. Date of interview: ____________________________
4. District: ___________________________________
5. Age: ______________________________________
6. Gender: ____________________________________

7: Working position of respondent: _________________

8: Duration in current position: ____________________

9: Education level: ______________________________

General opinions regarding the malaria surveillance system

1. How many years have you been working in the area of malaria? (Icebreaker question)

----

2. What is the current malaria situation in China?

----

3. What is the current situation of malaria surveillance system in China?

---

4. Could you please talk about the development of malaria surveillance system in China in last 10 years/(or since SARS outbreak in 2003) from your perspective?
(Prompts:
-Awareness
-Reporting timeliness/completeness/accuracy
-Surveillance capacity
-Technology
-Funding)

---

5. What aspect(s) do you think still need improvements regarding current malaria surveillance system?

General opinions regarding the 1-3-7 malaria surveillance strategy

1. Could you please introduce/explain the current Chinese malaria 1-3-7 surveillance strategy to me?

---

2. How about the current implementation situation of the malaria 1-3-7 surveillance strategy in China?
(Prompts:
-From the provincial level;
-From nationwide level;
-From different procedures of 1,3, and 7 procedures)

Specific operational aspects of the 1-3-7 malaria surveillance strategy

1. In which case a malaria case will be reported?
(Prompts:
-Suspected malaria cases (->then how to suspect a malaria case?)
-Confirmed malaria cases (->then by which method?)

---

2. Do you think there are any difficulties regarding reporting malaria cases?
(Prompts:
-If yes, please tell the detail of the difficulties
-If no, please tell what facilitators helped you on case reporting)

---

3. What are the recommended malaria diagnostic tools?
(Prompts:
-Microscopy
-RDT
-PCR)

----

4. What are the current using malaria diagnostic tools in your working station/place?

----

5. What do you think of this tool?
(Prompts: Aspects of sensitivity/cost/easy doing)

----

6. What do you think of the strategy regarding "malaria case confirmation within 3 days"?
(Prompts: aspects of feasibility/accuracy/costing)

----

7. Have you ever experienced difficulties regarding confirming malaria cases within 3 days?
(Prompts:
-If yes, please tell the detail of the difficulties
-If no, please tell what facilitators helped you on case confirmation within 3 days)

----

8. What do you think of the strategy regarding "classification of origin of malaria cases within 3 days"?
(Prompt: aspects of feasibility/accuracy/costing)

----

9. How do you differentiate a malaria case into imported or indigenous?

----

10. Have you ever had/experienced difficulties regarding classifying the origin of malaria cases within 3 days?
(Prompts:
-If yes, please tell the detail of the difficulties and are there any solutions?
-If no, please tell what facilitators helped on case classification)

----

11. If a malaria case was confirmed, then what wills you do in the next step?
(Prompts:
-Case investigation

-Or Evaluation of the risk of malaria foci
-Or Took actions in Focus investigation)

----

12. How to trace malaria patients, with a large mobilization (e.g. migrate workers and flowing population), in epidemiological investigations or conducting some public health actions?

----

13. For malaria patients with a large mobilization (e.g. migrate workers and flowing population), What do you think of tracing and investigating them malaria patients within 3 days?

(Prompts:
-What kinds of difficulties do you have/expect?
-What are the potential solutions?
-Feasibility/accuracy/costing/necessity)

----

14. How do you define an imported malaria case?

(Prompts:
-Definition: importation from endemic region of China or endemic countries?
-Reasons: travel history?)

----

15. What is a malaria focus?

----

16. What is a malaria focus investigation in the 1-3-7 strategy?

(Prompts:
-IRS
-Health Education
-RACD
-Specifically for migrant workers?)

----

17. In which condition the focus investigation (public actions) will be triggered?

Prompts:
-Imported malaria cases found?
-Indigenous malaria cases found?
-Active vectors?
-Cases occur during transmission seasons?
-Active foci?

----

18. Are there any indicators to define an active focus? If so, what is it?

----

19. What are public actions in active foci?
(Prompts:
-IRS?
-RACD?)

19.1 For IRS, could you please share the current guidelines?
(Prompts:
-How to spray (To which extent/To how large radius)?
-Who should responsible for the spraying?)

19.2 Are there any difficulties regarding IRS?
(Prompts:
-Hard to define the radius?
-Costing?
-Acceptability?
-Quality of the work?)

19.3 Are there any lessons learned regarding IRS?
(Prompts:
-Logistics?
-Improvement of acceptability?
-Quality control?)

19.4 For RACD, could you please share the current guideline?
(Prompts:
-What is demographic RACD?
-What is geographic RACD?
-To how large radius/At what time should RACD be conducted?
-Who should be responsible for the RACD?

19.5 Are there any difficulties regarding conducting the RACD?
(Prompts:
-Hard to define the radius and timing to do RACD?
-Costing?
-Acceptability?

-Quality of the work?)

19.6 Are there any lessons learned regarding conducting RACD?
(Prompts:
-Logistics?
-Improvement of acceptability?
-Quality control?)

----

20. What do you think of the timeframe (i.e., 7 days) to conduct focus investigation?
(Prompts:
-Remote areas?
-Transportation?
-Availability of health care staff?
-Hard to follow population?
-Costing?
-Lack of guidelines?
-Lack of experiences?)

----

21. If any above or new difficulties mentioned, then are there any solutions from your opinion?

----

22. What do you think of the feasibility of the timeframe regarding the 1-3-7 procedures?

----

23. What lessons/experiences will you think important regarding the overall implementation of 1-3-7 approach?

Communication of 1-3-7 between county level and provincial level

1. County level CDCs are the basic responsible units of the the 1-3-7 surveillance. How do you think about the capacity of county CDCs for the implementation of the “1-3-7 surveillance strategy”?

----

2. Under which condition the provincial CDC/institute will help/assist the county CDC for the implementation of “1, 3, 7 surveillance strategy”?

----

3. What assistances have you been requested from the county/township CDC on the implementation of the current 1,3,7 surveillance strategy?
(Prompts:
-Diagnosis
-Epidemiological investigation)

----

4. How you think the communication regarding requesting/providing data and information in the 1-3-7 surveillance between provincial level and county level?

----

5. Is there any gaps regarding requesting/providing help between provincial level and county level?

Yes ☐ No ☐

5.1 If yes, what kind of gaps?
(Prompts-communication/policy/conflict of interest)

5.2 If no, what are the lessons/experiences?

Promotion of the 1-3-7 approach nationwide

1. From your point of view, what do you think of promoting 1,3,7 surveillance strategy nationwide?

----

2. What kind of strategies do you think could be useful for promoting this strategy nationwide?
(Prompts: By law? Providing incentives?)

The 1-3-7 surveillance strategy and the national goal

1. What is the role of 1,3,7, malaria surveillance strategy in elimination program?

----

2. What do you think of the national malaria elimination goal in China by 2020?

----

3. What aspects do you think need to be strengthened to achieve elimination goal?

Thank you for your time and answers!
