# Supplementary material for: Challenges in and lessons learned during the implementation of the 1-3-7 malaria surveillance and response strategy in China: a qualitative study
Source: Infect Dis Poverty. 2016 Oct 5;5:94. doi: 10.1186/s40249-016-0188-8 (PMC5050603; doi:10.1186/s40249-016-0188-8)
Supplement: Additional file 4: Table S1. — Selected supporting quotes about challenges and lessons learned in case reporting within 1 day. Table S2. Selected supporting quotes about challenges and lessons learned in case investigation within 3 days. Table S3. Selected supporting quotes about challenges and lessons learned in focus investigation within 7 days. Table S4. Selected supporting quotes about challenges and lessons learned from the entire 1-3-7 strategy. (PDF 92 kb) [file 40249_2016_188_MOESM4_ESM.pdf]

Additional table 1. Selected supporting quotes of challenges and lessons learned in case reporting within one day

**Challenges**	Selected supporting quotes
Inadequate surveillance capacity of primary health staff	*R: "We are afraid that the patients was delayed by the village doctors and do not come to us in the first time. I am worried about this access delay, because it [malaria] now became less and less, and the village doctors might also forget about it and will not suspect it."（#37-county level）*
Delay of presence of malaria patients in clinics	*R: "Some time ago, we faced to a malaria patient who was back from Africa countries, but he and his families all wanted to hide this abroad history and purposefully told us they were back from Singapore. They are afraid of discrimination and therefore hide the working experience."（#16-Province Level）*
Inadequate diagnostic tools	*R: "The province [CDC] bought RDTs and distributed them into different health care centers, but the coverage is limited and the diagnosis of malaria cannot totally rely on it [RDTs]." (#17-province level)*
Village clinics are not covered under the reporting system	*R: "Malaria reporting system has already rooted deep into township levels, which means the heath staff from township health centers could direct report malaria cases through the malaria reporting system. However, the most primary health care level is still missing, which is village. Village [village doctors] is actually the most primary health care provider in China. However, it is also a big challenge to guarantee the universal accessibility and coverage of computers and Internet at village levels."(#16-province level)*
**Lessons learned**	Selected supporting quotes
Continues capacity building at primary health clinics	*R: "We have several regular trainings every year. It includes many aspect of malaria like policies, working requirements, and evaluation standards. At the same time, the technical staff was trained on the examination of plasmodium." (#30-county level)*
A well-functioning surveillance infrastructure	*R: "We have built a very well-functioning reporting platform. No matter from where you report a malaria case, the health staff in charge of will immediately receive a text message through the mobile phone." (#05-city level)*
Technical flexibility of suspected cases reporting	*R: "If a reported suspected malaria case is not true, we just delete it and it will be no any blame of reporting it." (#16-province level)*

Additional table 2. Selected supporting quotes of challenges and lessons learned in case investigation within three day

**Challenges**	Selected supporting quotes
Respecting the 3 days timeline	*R: "Patients visited doctors at outpatient and left, and usually their contact information was recorded by doctors. The doctors are not in charge of epidemiological investigation and they [doctors] will contact us. But when we contacted the patient afterwards, there is a problem of losing of follow up. For example, there is a patient returned from abroad and was suspected as malaria [by the local hospital]. We were informed and contacted the patient on the next day, and he already went to another province to see doctors. So we reported this case because his census registered here, but we could not do epidemiological investigation." (#13-province level)*<br>*R: "Sometimes health staff from primary health centers sent samples to the province for confirmation. Considering the delivery process, the working time and the distance, could the samples definitely be received and confirmed in three days remains a question."(#16-province level)*
Difficulties of species identification	*R: "Species classification is difficult for us. Moreover the microscopy now we have is from 1980s and sometimes it sees not very clear." (#08-county level)*
Subjective and technical aspects of case classification	*R: "We rely on the epidemiological investigation to judge the origin of a reported malaria case (imported or indigenous). For falciparum malaria, it may work fine…… However it may be difficult to apply in vivax malaria. For example, it is hard to say whether the P. vivax patient is an imported malaria case or a relapse case of indigenous malaria with long incubation time." (#18-province level)*<br>*R: "Although we could rely on the molecular biology technology to distinguish a malaria case originally from African or China, we could not distinguish a malaria case is from Myanmar or China. They are very close related and they even are same population from genetic perspective." (#16-province level)*
Limited quality control of case investigation	*R: "It is hard to say how well it [case classification] has been done." (#17-province level)*

**Lessons learned**	Selected supporting quotes
The importance of information technology (IT) support	*R: "We sent blood samples which we are not sure to the province level for further confirmation. I remembered when we suspected the first case this year in February, which occurred during The Spring Festival, and we took photos from the microscopy lens of the slide and sent to the province by mobile phone message. Although no buses at that time as people are all in holiday, we got quick responses from province CDC through this way." (#08-county level)*
New research findings	*R: "In the process of confirmation of low parasitemia, new sensitive diagnostic tool are under investigation. For example, the LAMP may help to solve the diagnostic difficulties of low parastemia". (#18-province level)*

Additional table 4. Selected supporting quotes of challenges and lessons learned in focus investigation within seven day

**Challenges**	Selected supporting quotes
Complexity and difficulties evaluation of local transmission risk	*R: "A foci caused by imported falciparum malaria in malaria non-endemic areas do not need to do vector control activities (spraying), because there are no active vectors; If it occurred in areas with active vectors, we need to do vector control activities. But we still do not have enough laboratory evidence to prove that the vectors in our province are not susceptible to the imported falciparum malaria species at all."(#18-province level)*
Respecting the 7 days timeline	*R: "For the areas not very far away, it is very quick for us to do the focus investigation. But the returning migrant workers usually bought another apartments/houses in the county or city [they register in the village]. In this case, although it is reported from where the citizenship registered, the living places could be not the same. Therefore it might be a delay." (#37-county level)*<br>*R: "In our province, some villages are not documented (coded) in the reporting system. It may happen that the health centers did the work but they cannot find the exact village code in the system. In this case I could only ask them [health staff] to collect information, send to the province and reported from the province." (#01-province level)*
IRS and RACD in potential active/active foci	*R: "The radius of spraying is ambiguous and usually evaluated by observation of the health staff. For example we spray the families with a circle with a radius of 50 meters. (#33-county level)*<br>*R: "In practice, how large radius we should spray was depend on actual circumstances. For example, if a foci occurred in high-populated region, we spayed all the residents with a radius of 150 meters. (#30-county level)*<br>*R: "In my opinion, spraying the neighbours of index case is more easier to understand and to operate by the health staff......nevertheless, a regulated raidus like 80 meters or 100 meters is not easy to implement." (#14-province level)*

Acceptance of actions by population	*R: "The documents regulated how to spray one room. When we actually went to the patient's house, the rooms [in the village] are not like in the city which separated into eating room, sleeping room and so on. It is common that some foods stored in the bedroom. I remembered all the clothes just hang on the wall, and we cannot spray the clothes. In this case, we actually need firstly to clean up the whole room if we want to conduct spraying carefully…… Moreover some people would not like strangers to touch their property.." (#41-township level)*<br>*R: "Now I am afraid of conducting spraying activities. A few days ago, we prepared to conduct spray in one patient's house in our township. We went to there in the morning and the families is not agree with us for spraying. We explained until noon and they are still not agreed, and without agreement we cannot do this. " (#25-village level)*
Limited quality control of focus investigation	*R: "The spraying was done mostly by the villager doctors. We asked them to spray the room inside, the pigsty, the rodeo and the toilet. But if you ask the quality of the work, actually I also doubt because they [village doctors] are all very busy." (#11-township level)*
**Lessons learned**	Selected supporting quotes
Logistical aspects	*R: "It is important to make appointments with patients before going to their home place to take actions."(#25-village level)*
Community acceptability	*R: "There are some aspects you need to be careful to do focus investigation. For example, for the family with big fishponds, we may spray the surrounding areas of the pond and we will never spray small fishponds or inside of ponds. Before spraying, it is important to let the villagers know that their fish will not be harmed. For another example, when we spray the room inside, the villagers should all go out and all expensive furniture should be covered in order not to be sprayed and we only sprayed the wall." (#27-township level)*<br>*R: "Some people in the same village will ask us whether the patient had some serious diseases. We will not answer and keep privacy of the patient. We are afraid that the families of the patients and the patients will be discriminated (by other villagers)." (#28-village level)*

Methods of quality control	*"Now when the health staff finished a malaria foci investigation, they are required to fill in a form and also keep and sent the photos of the process of focus investigation, which could show the process of spraying or doing something to the county level. Because we are afraid that the health staff is not done the work or not done well."(#23-village level)*
Active screening of the migrant workers and their peers upon return to China	*R: "We will find out from which company workers have been migrated. In the past in our county there were about 40 export labors companies, we tried very hard to keep in contact with them. But many of them [companies] ignored us until the incidence of death cases and they compensated for it. After this [the occurrence of death cases], they [companies] started to be collaborative. Now we tried to get the name lists of the coworkers who returned at the same time with the case."(#30-county level)*

Supplement table 4. Challenges and lessons learned of overall malaria 1-3-7 surveillance strategy

**Challenges**	Selected supporting quotes
A lack of motivation of primary health staff	*R: "I do not know the effect of spraying. It feels me that if we do not do the spraying, then there are also no malaria cases. So the effect [of spraying] seems to be not so obvious." (#40-village level)* *R: "Although the work is our duty, I feel it is somehow difficult that we ask them (village doctors) to do the work as we do not pay them. After all it is not their work and they just assisted us. If we work like this way for long term and I think they (village doctors) will emotionally resistant." (#39-township level)*
Increasing returning migrant workers from malaria highly endemic countries	*R: "Most returning migrant workers understood and know that our work is good for them so they will collaborate with us, but things are not always like this. In this year, for example, there was a malaria patient [imported case] and we went to his home for at least 4 times and he is not willing to go to hospital as he regarded it is not a big deal to have malaria…… The export labor companies gave the workers some drugs before the workers prepare to return back and told them just to take drugs when they developed fever." (#30-county level)*
The complexity to establish multi-sectors collaboration	*R: "We left our QQ number [Chinese social communication media] to them [foreman in the export labor company]. We requested a full name list of migrant workers exported through their companies. But the foreman/company postponed the request from today to tomorrow, from tomorrow to next month. The reason is the boss of the [export labor] company had difficulties to enroll new workers because we conducted a lot of propaganda. Therefore they [foreman in the export labor company] rejected to provide us the name lists." (#11-township level)*
**Lessons learned**	Selected supporting quotes
High government commitment	*R:"We carefully supervise case investigation procedures. When we noticed one reported case from the reporting system, we will call the responsible health units and ask them to conduct the case investigation as soon as possible." (#02-province level)*
Aspects better targeting and	*R: "We contacted the foreman and told them [foreman] that you [foreman] brought these workers abroad and they [worker] got malaria and malaria cannot be treated to totally recovery just in one or two days, so we do*

management of returning migrant workers	*not have enough stock of the drugs if many patients returning back at the same time. We explained to them [foreman] that we need to transport anti-malaria drugs from the city CDCs here and it took time. Therefore we need to do registration of the migrant workers in order to store enough drugs. Through this way, we could get the name lists [of export labors]." (#10-township level)*
Financial incentives	*R: "Completed reporting of a malaria case will get financial awards (200 Yuan) in our province. We are now considering establishing awards also for sending malaria blood samples." (#18-province level)*
Continues education and training	*R: "The only goal of Export Company is to earn money. It is necessary to offer intensive education to both [people in charge of] companies and migrant workers. Since 2009 we launched the campaign called "A letter to migrant workers". Through it migrant workers will be educated on many malaria related aspects before they went abroad."(#30-county level)*<br>*R: "We have several regular trainings every year. It includes many aspect of malaria like policies, working requirements, and evaluation standards. At the same time, the technical staff was trained on the examination of plasmodium." (#30-county level)*
